# Multiple plexiform schwannomas in the plantar aspect of the foot: case report and literature review

**DOI:** 10.1186/1471-2474-15-342

**Published:** 2014-10-11

**Authors:** Xiao-na Li, Jian-ling Cui, Seemon Petrus Christopasak, Abhinav Kumar, Zhi-gang Peng

**Affiliations:** Department of Radiology, The Third Hospital of Hebei Medical University, Hebei Province Biomechanical Key Laborary of Orthopedics, Ziqiang road 139, Shijiazhuang, Hebei 050051 China

**Keywords:** Plexiform schwannoma, Neurofibromatosis, Magnetic resonance imaging

## Abstract

**Background:**

Plexiform schwannoma (PS) is a rare, peripheral nerve sheath tumor arranged in a plexiform pattern.

**Case presentation:**

We report an unusual case of a 19-year-old woman, who complained of pain in the plantar aspect of the left foot. Magnetic resonance image (MRI) demonstrates three solitary nodules of varying sizes in the deep soft tissue of the plantar aspect of the foot that are homogeneously isointense to skeletal muscle on T1-weighted images and hyperintense on T2-weighted fat-suppressed images, especially the rim of the lesion. Subsequent pathological examination confirmed the diagnosis of PS.

**Conclusion:**

MRI characteristic plays an important role in detecting this rare lesion. A review of the literature on PS is also presented.

**Electronic supplementary material:**

The online version of this article (doi:10.1186/1471-2474-15-342) contains supplementary material, which is available to authorized users.

## Background

Schwannoma is a benign, peripheral nerve sheath tumor, occurring commonly in the persons of 20–50 years old [1], and manifests about 5% of benign soft-tissue neoplasm [2]. Schwannomas usually involve in head, neck, flexor surfaces of extremities and nerves [2, 3]. Plexiform schwannoma (PS) is a rare distinctive variant, accounting for 2-5% of all schwannomas [4–6]. It frequently affects patients aged 30–40 years with no sex predilection, and occurs commonly in the dermis and subcutis with a predilection for head and neck [7]. Up to now only 11 cases located to the foot have been reported and published in English medical literature [8–11]. Multiple PSs in the plantar aspect of the foot are very rare. The diagnosis of PS mainly depends on histopathologic and immunohistochemical features. The MR imaging features of PS, however, were described only partially by some case reports [12]. If the imaging features can be recognized, it would be the helpful for preoperative diagnosis. The current paper aims to demonstrate distinct imaging features of PS in the plantar aspect of the foot which may be vital to facilitate the diagnosis process in the future and to give a short literature review.

## Case presentation

**Figure 1:**
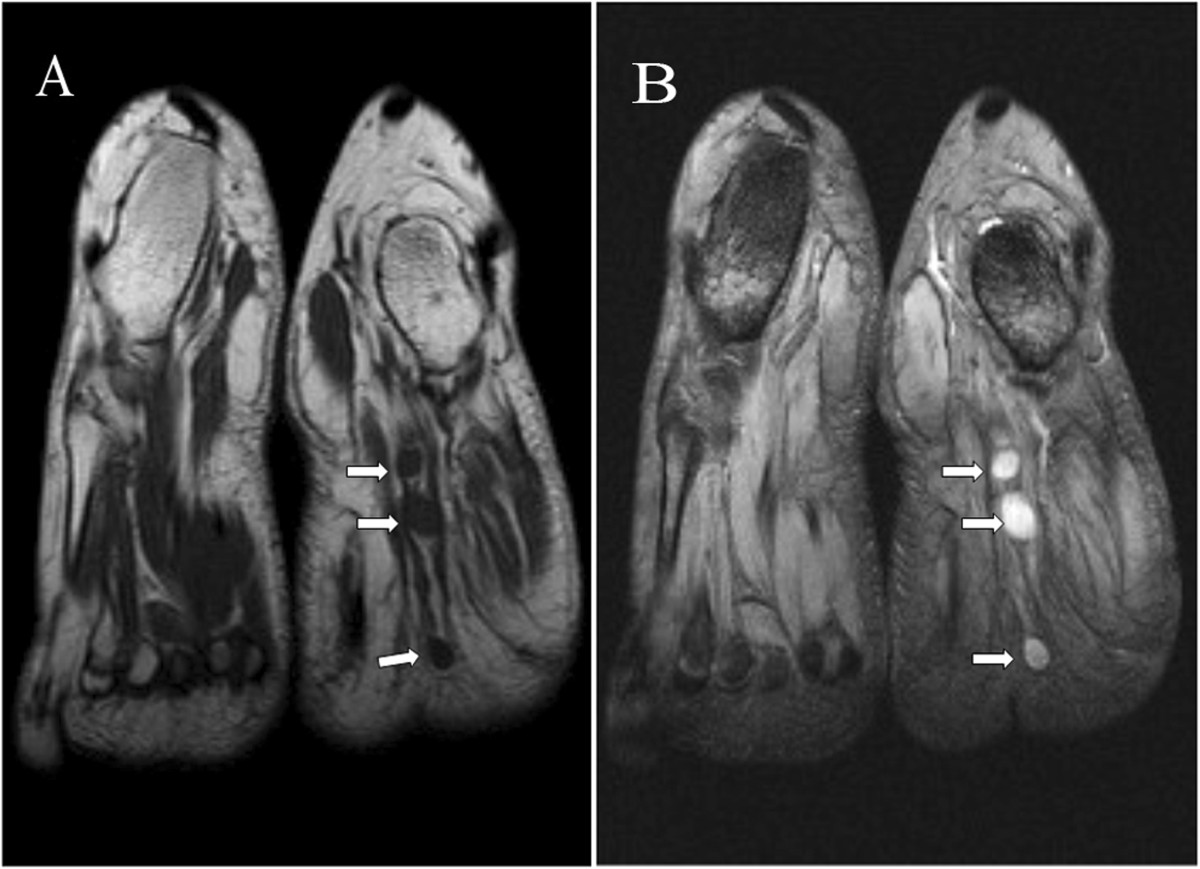
**Coronal MR images showing linear round lesions. (A)**: T1-weighted fast-spin echo image (TR/TE: 512/11) shows the lesions with homogeneous isointensity to skeletal muscle (*arrows*). **(B)**: T2-weighted multi echo data image combination sequence (TR/TE: 865/23) demonstrates three prominent nodules of high intensity signal (*arrows*).

**Figure 2:**
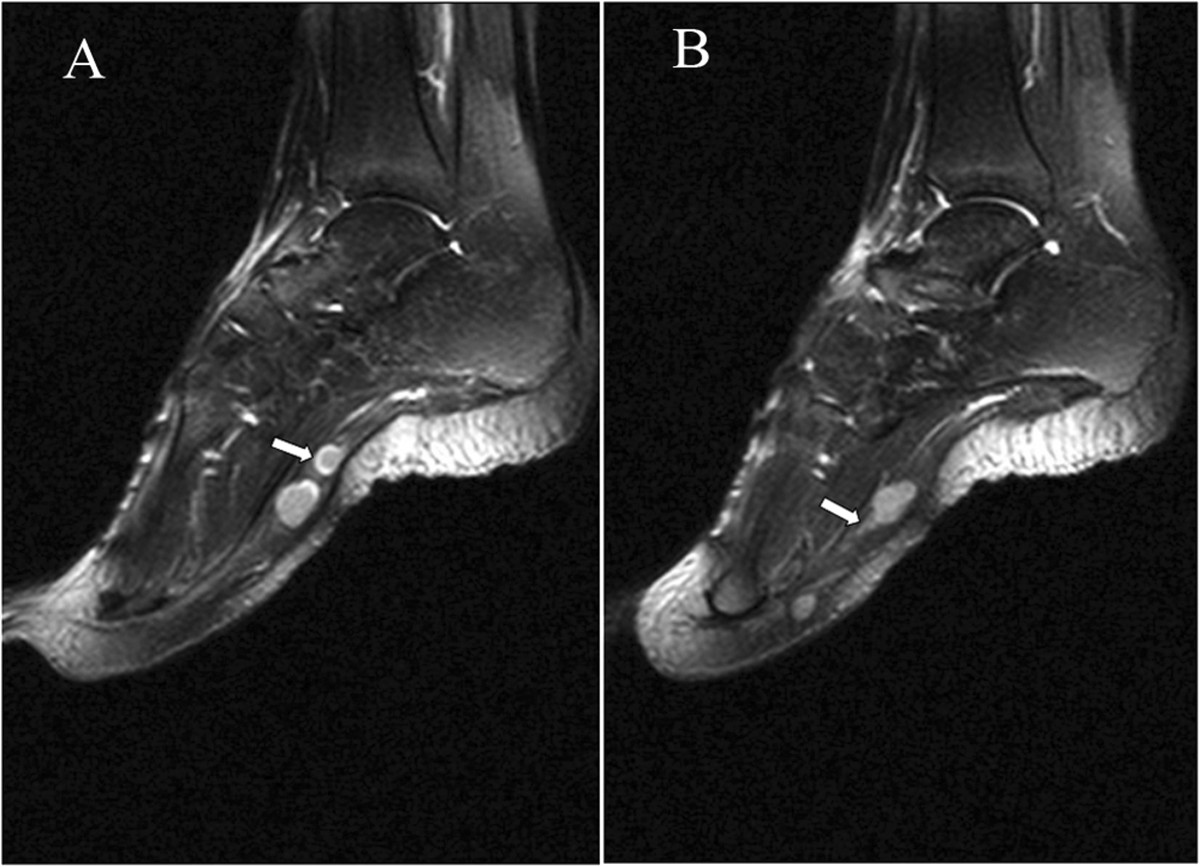
**Sagittal T2 fast spin-echo-weighted fat-suppressed image (TR/TE: 3000/40, TI: 150) shows nodules localized in the plantar aspect of the foot. (A)**: The rim of the tumor shows hyperintense signal in comparison with inside of the tumor (*arrow*). **(B)**: The surrounding nerve seems like a mouse-tail (*arrow*).

**Figure 3:**
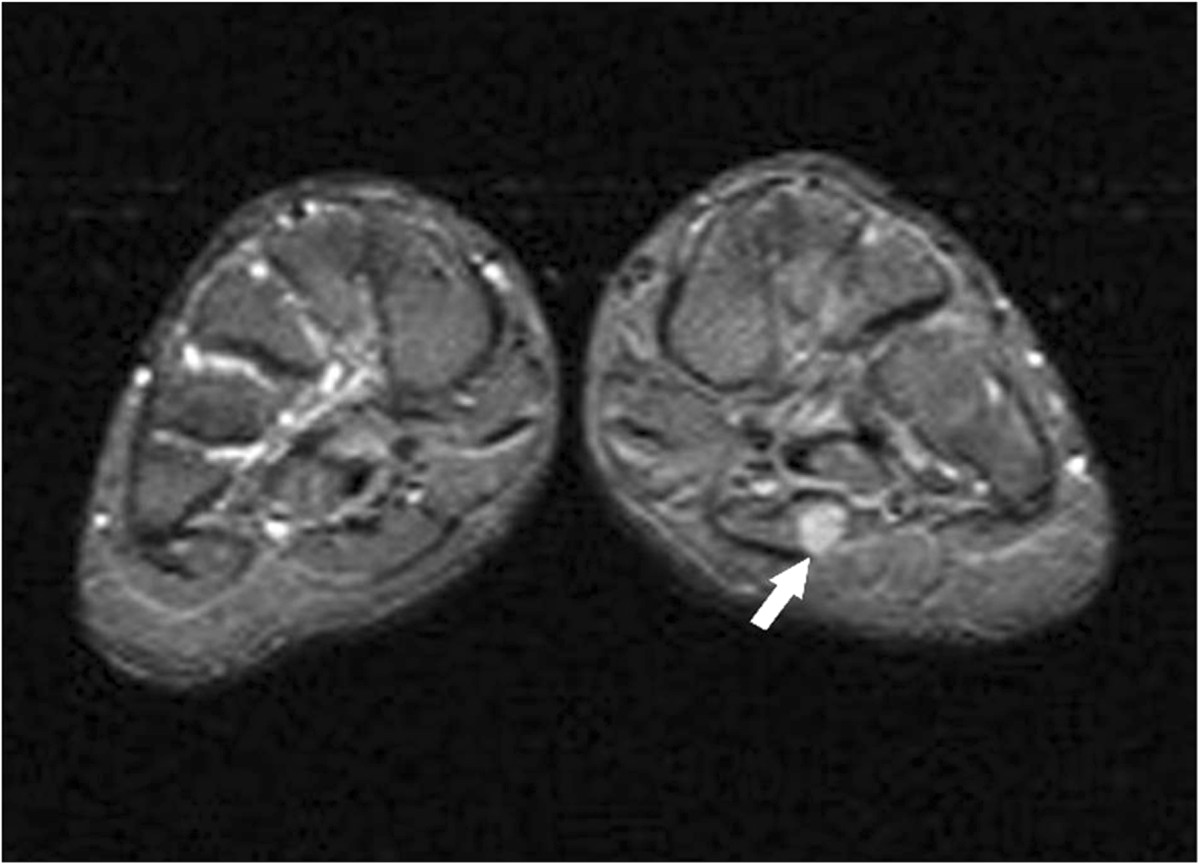
**Axial short T2 turbo inversion recovery (STIR) image (TR/TE: 4500/26, TI: 130) shows the nodule located in flexor digitorum brevis below the plantar aponeurosis with high signal (arrow).**

**Figure 4:**
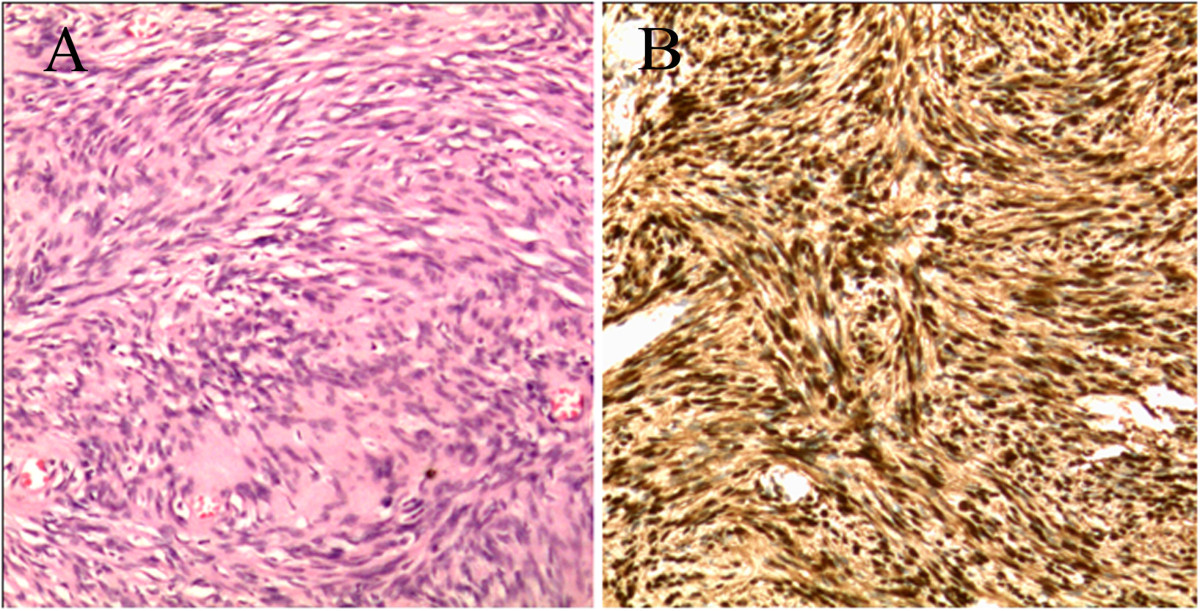
**Photomicrograph of the tumor. (A)**: Microscopic specimen of the lesion shows long spindle-shaped tumor cells and nuclei palisading with multiple fascicles (Hematoxylin-eosin, H & E, original magnification × 200). **(B)**: The tumor cells stain positive for S-100 protein and exhibit strong immunoreactivity for S-100 protein (Original magnification × 200).

A 19-year-old woman complained of pain in the plantar aspect of the foot for eighteen months. The pain was moderate and tolerable in the initial stage, and often worse at night, and even much worse with compression and low temperature. The patient visited the department of radiology due to aggravation of the local pain for the last four months. Physical examination revealed three prominent soft tissue nodules with swelling and tenderness, in the plantar aspect of the left foot, but without adhesion to the surrounding tissue. Neurological examination showed numbness in the medial aspect of the distal plantar aspect of the left foot. The movement and strength of both toes and foot were normal. The patient denied any history of trauma. The family history of neurofibromatosis type 2 (NF-2) was also negative.The MRI examination was performed to characterize the nodules detected during physical examination. On MR imaging, a linear nodular distribution of the lesions, located in the mid part of the plantar aspect of the left foot was observed (Figures 1 and 2). The lesions were homogeneously isointense to skeletal muscle on T1-weighted images (Figure 1A) and hyperintense on T2-weighted fat-suppressed images (Figures 1B, 2 and 3). No evident cystic degeneration was observed on all the MR images. The rim of the lesion appeared as hyperintensity on T2-weighted fat-suppressed images (Figure 2A). A small extension of high signal arising from the lateral aspect of the lesion seemed like a mouse-tail (Figure 2B). Two of three nodules were located in the flexor digitorum brevis below the plantar aponeurosis (Figures 2A and 3) and one nodule was in the superficial fascia of the third metatarsophalangeal joint (Figure 2B). These nodules were not scattered and they had a peculiar linear distribution originating from the branches of the common plantar digital nerves. No invasion of the surrounding tissue was observed. Based on the clinical and imaging findings, they were initially thought to be glomus tumor, neurofibroma or schwannoma. Multiple PSs were suspected and surgical excision was recommended.During the operation, there were three round nodules located in the subcutaneous tissue, between the skin and deep fascia, with the sizes of 0.5 × 0.5 × 0.8 cm, 0.7 × 1.0 × 1.2 cm and 1.0 × 1.1 × 1.5 cm, respectively. The nodules were elastic and movable. These nodules were not well demarcated from surrounding peripheral nerve tissue. Intraoperative frozen sections of the nodules were performed and the examination indicated PS. Therefore excision of the lesions was performed. Histopathological examination with the use of the hematoxylin-eosin staining demonstrated that the specimen was composed of uniform spindle cells. Characteristic cellular Antoni-A areas with nuclear palisading and Verocay bodies were observed (Figure 4A). The tumor cells stained positive for S-100 protein and exhibited strong immunoreactivity for S-100 protein (Figure 4B). The pathological findings give the diagnosis of PS in the plantar aspect of the foot.

After operation, the patient reported hypoesthesia in the distant third and fourth toes. The patient was followed up for two and a half years postoperatively, and there was no evidence of local recurrence or new lesions at other parts of the body.

## Discussion

PS is a rare variant of schwannoma [5]. It occurs mostly as a solitary lesion in the skin or subcutaneous tissue, or uncommonly located in the deep soft tissue [13]. Most PSs are small with a maximal diameter less than 2 cm, originating from the superficial nerves. Trauma may play an etiological role in the formation of this lesion [14, 15]. Since up to 5% of cases are believed to be associated with NF-2 [3, 10, 16–19], careful intracranial and spinal MR examinations are required to exclude the potential NF-2 in young patients [20].

MRI is a useful tool in detecting soft tissue neoplasms. MRI is also used in the diagnosis of multiple PSs [10, 11]. Grossly, the rim of the PS nodule is smooth. The MRI features of this tumor are similar to peripheral nerve sheath tumors including heterogeneous hypo- or isointensity compared with surrounding muscles on T1-weighted MR images, and heterogeneous hyper- or isointensity to subcutaneous fat on T2-weighted MR images [21–23]. It is reported that multinodular growth pattern in a single lesion and evident cystic degeneration on T2 weighted image are the characteristics of PS, which is helpful in the differential diagnosis [24, 25]. However, confusing results were reported. Ikushima et al. [8] failed to delineate the multinodular architecture in PS of the foot based upon the preoperative CT and MRI. Multinodular pattern was not also observed in the current case. Therefore, we present this rare case as an effort to further characterize the lesion and to facilitate the diagnosis of similar cases.

In the current study, MRI showed three round-shape nodules located in the plantar aspect of the foot. These nodules were not scattered and they had a peculiar linear distribution originating from the branches of the common plantar digital nerves. The MR signal of these nodules was homogeneous, maybe partially due to the small size of the nodule. No evident cystic degeneration was noted in any of the three lesions, which were different from conventional schwannomas. The mass of the solitary nodule was surrounded by a hyperintense rim on T2-weighted images with fat suppression, which have not been reported and required further investigation to identify the reason. Particularly, the surrounding nerve of the tumor appeared like a mouse-tail, which implied the neurogenic tumor.

It is important to differentiate PS from plexiform neurofibroma, because there are some similarities in clinical and imaging characteristics of them. In contrast to PS, plexiform neurofibroma originating from peripheral nerves usually occurs in early childhood with a positive family history of neurofibromatosis [4, 26, 27], and has a significant risk of malignant transformation (4%), which has not been reported to occur in PS [26, 28].

Some authors reported [18, 29] that plexiform neurofibroma is similar to PS that presents on imaging with intermediate signal intensity to the skeletal muscle on T1-weighted images and hyperintensity on T2-weighted images. However, there are some imaging features which can distinguish PS from plexiform neurofibroma. Plexiform neurofibromas are essentially pathognomonic for NF1 with diffuse involvement along a nerve segment or its branches, giving a “bag of worms” appearance [30, 31]. Few flow voids within the lesion are displayed on T2-weighted images of plexiform neurofibromas [29]. Although post-contrast T1-weighted image was helpful to make a differential diagnosis, Shinde et al. [18] reported that post-contrast images could not make a definite diagnosis for the various pattern of the nodule [32]. No further contrast examination was done in the current study. Histopathologic findings are helpful in differentiating PS from plexiform neurofibroma definitely.

The histopathologic differential diagnosis of PS includes plexiform neurofibroma and plexiform fibrohistiocytic tumor [24], which requires wide excision to avoid potential recurrence and metastasis [5]. PS consists of multiple intradermal or subcutaneous nodules composed primarily of cellular Antoni A regions with nuclear palisading and verocay bodies [30]. But plexiform neurofibroma usually does not have these characteristics [24]. In contrast to conventional schwannomas, PS grows in a plexifrom pattern. It consists of multiple interlacing and interconnecting fascicles and nodules usually with the Antoni A features of a solitary schwannoma [33] but without Antoni B zones. Nodules were full of slender spindle cells with nuclear palisading and plexiform and spiral arrangement, especially, consisting of verocay bodies. Immunohistochemical studies showed the tumor cells stained positive and exhibited strong immunoreactivity for S-100 protein.

### Limitations of the study

Because the study was a retrospective study, no contrast MR examination was done and intraoperative photos were not acquired. Thus it must be assumed that the outcome of this case report would be more worth to study.

## Conclusions

We present a case of multiple PSs in the plantar aspect of the foot. In contrast to conventional schwannomas, multiple PSs are rare, and the imaging features of this lesion include the homogeneously hyperintense signal without multinodular and cystic degeneration on T2 MRI. We report this case with an effort to describe its MRI characteristics for diagnosis and also hope to provide informative data for further comprehensive analysis.

### Consent

We conform that the patient has given his written consent for the case report to be published. A copy of the signed consent is available for review upon request.

### Ethical Board Review statement

This material has not been published and is not under consideration elsewhere. This study receives no financial support.

## Authors’ original submitted files for images

Below are the links to the authors’ original submitted files for images. Authors’ original file for figure 1 Authors’ original file for figure 2 Authors’ original file for figure 3 Authors’ original file for figure 4 Authors’ original file for figure 6 Authors’ original file for figure 7
