# Supplementary figures and images for: Multiple plexiform schwannomas in the plantar aspect of the foot: case report and literature review
Source: BMC Musculoskelet Disord. 2014 Oct 11;15:342. doi: 10.1186/1471-2474-15-342 (PMC4210527; doi:10.1186/1471-2474-15-342)

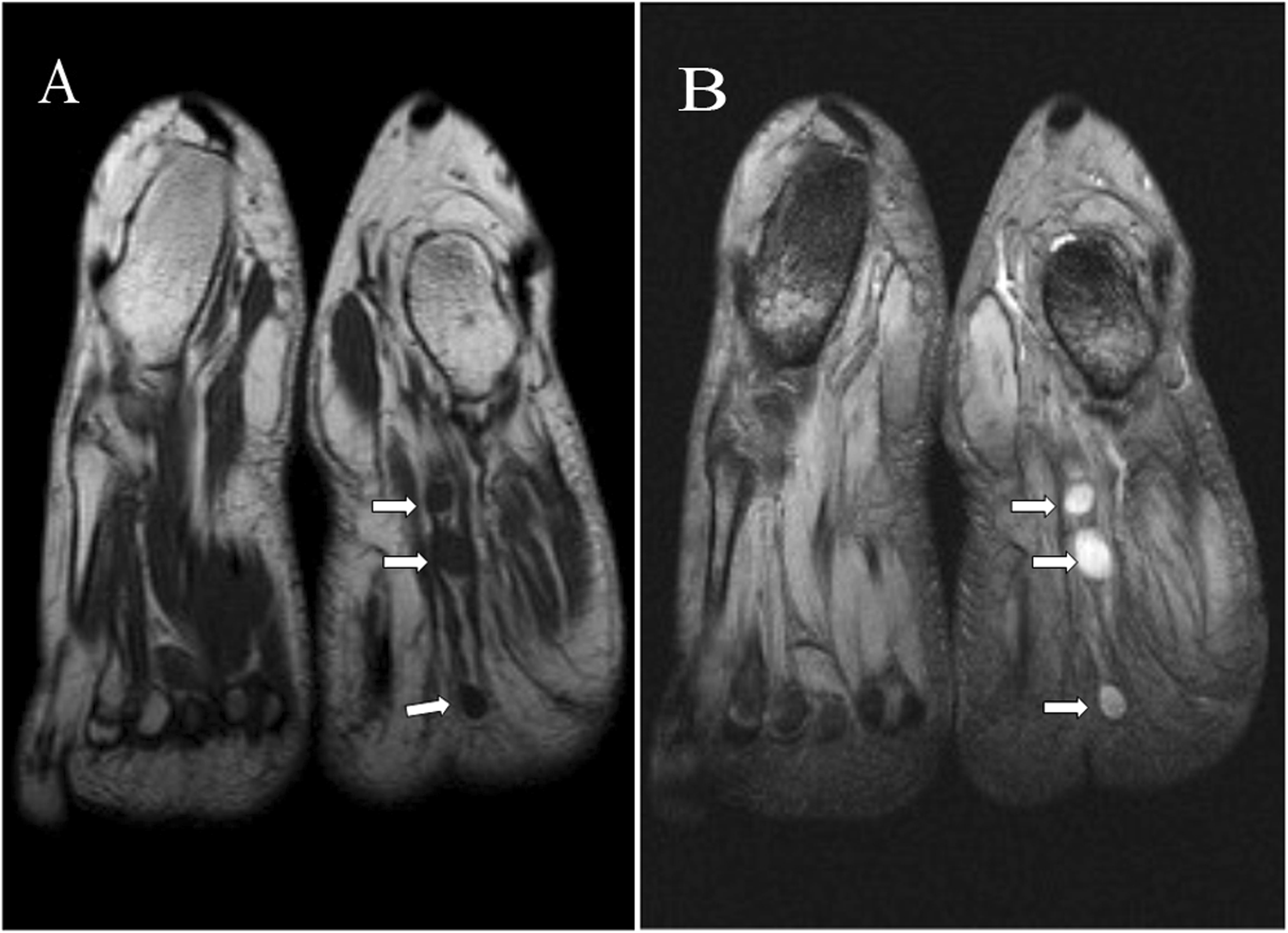

Supplement: Supplementary file 1 — Authors’ original file for figure 1 [file 12891_2014_2282_MOESM1_ESM.tif]

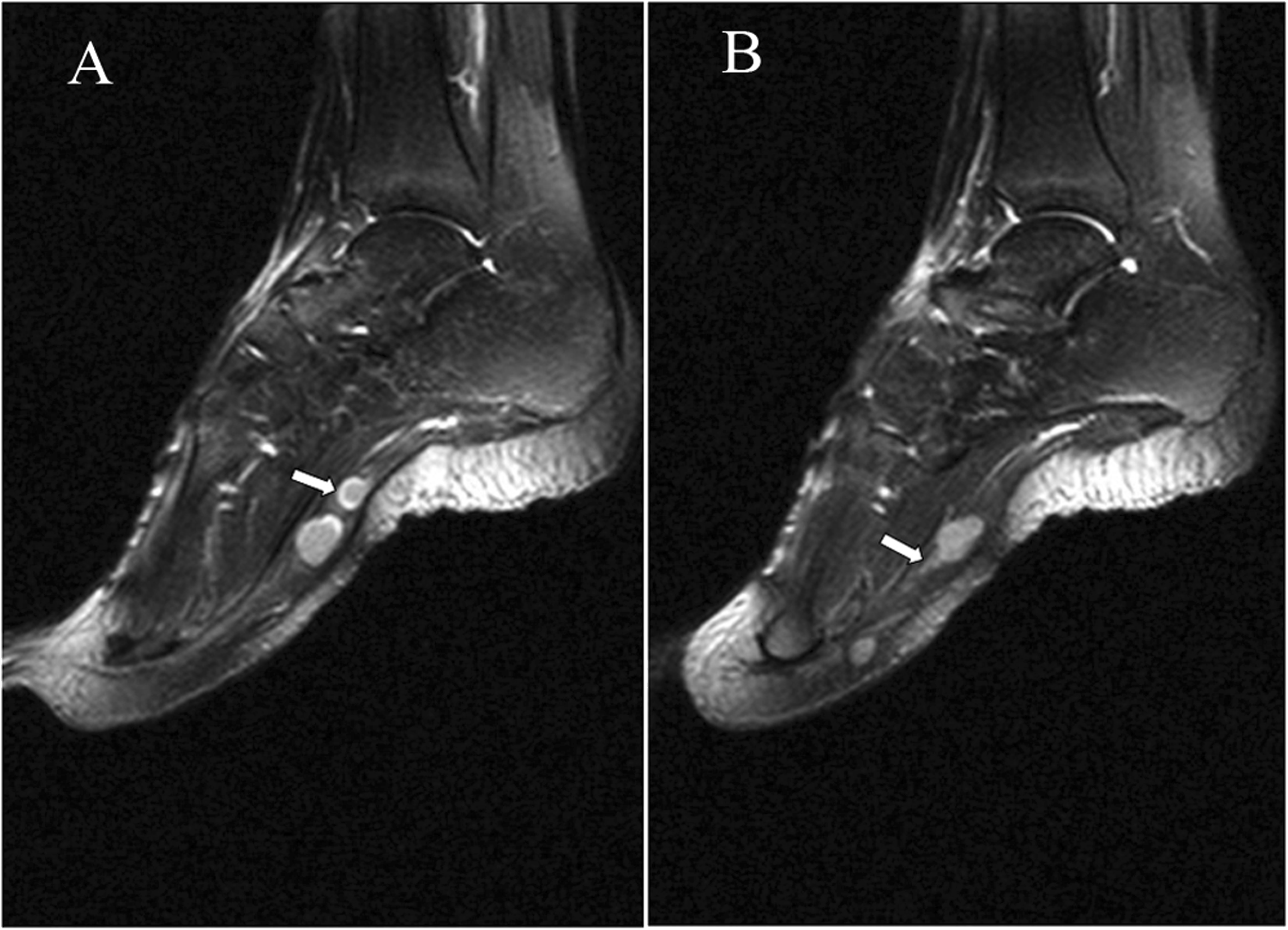

Supplement: Supplementary file 2 — Authors’ original file for figure 2 [file 12891_2014_2282_MOESM2_ESM.tif]

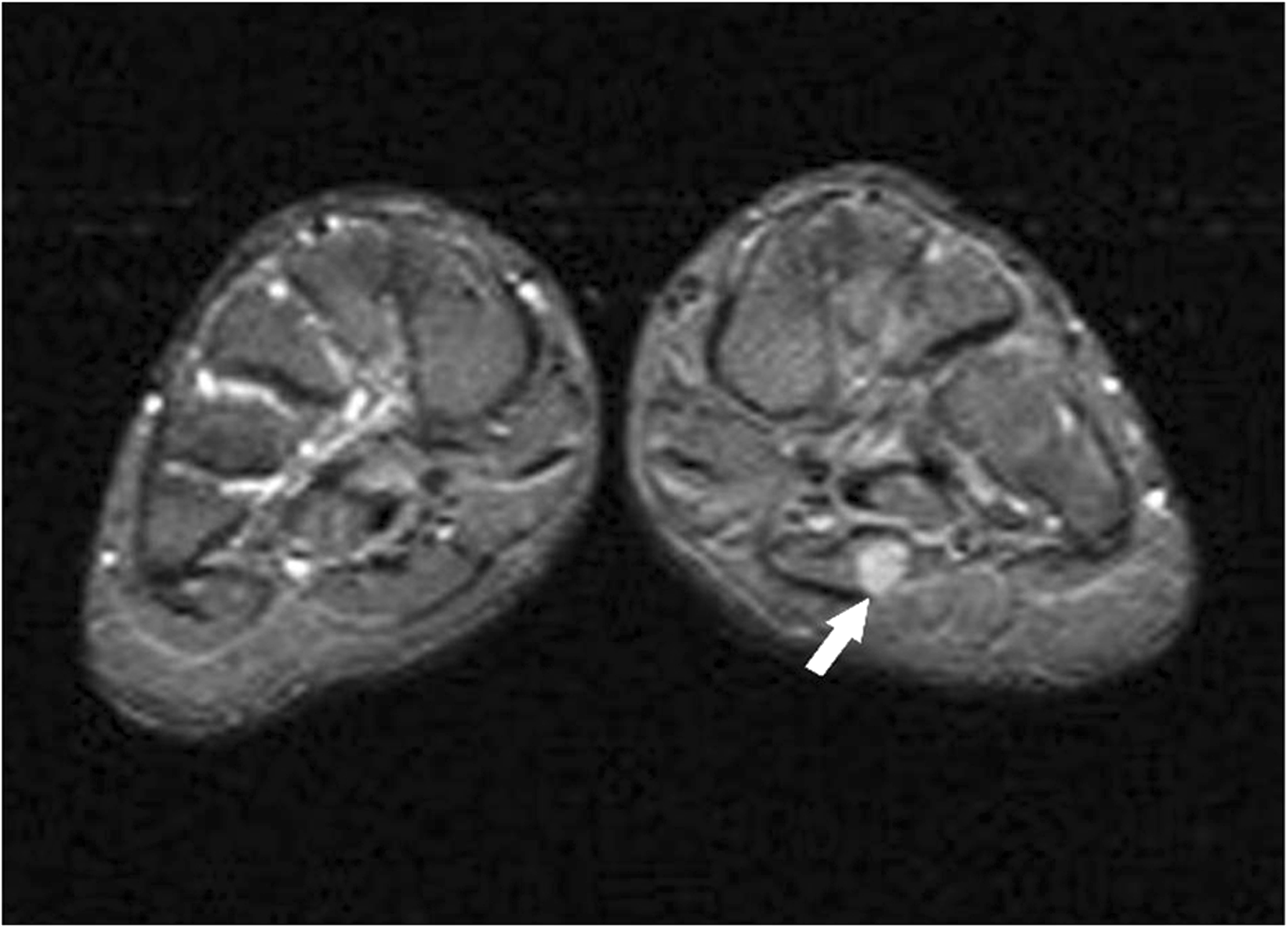

Supplement: Supplementary file 3 — Authors’ original file for figure 3 [file 12891_2014_2282_MOESM3_ESM.tif]

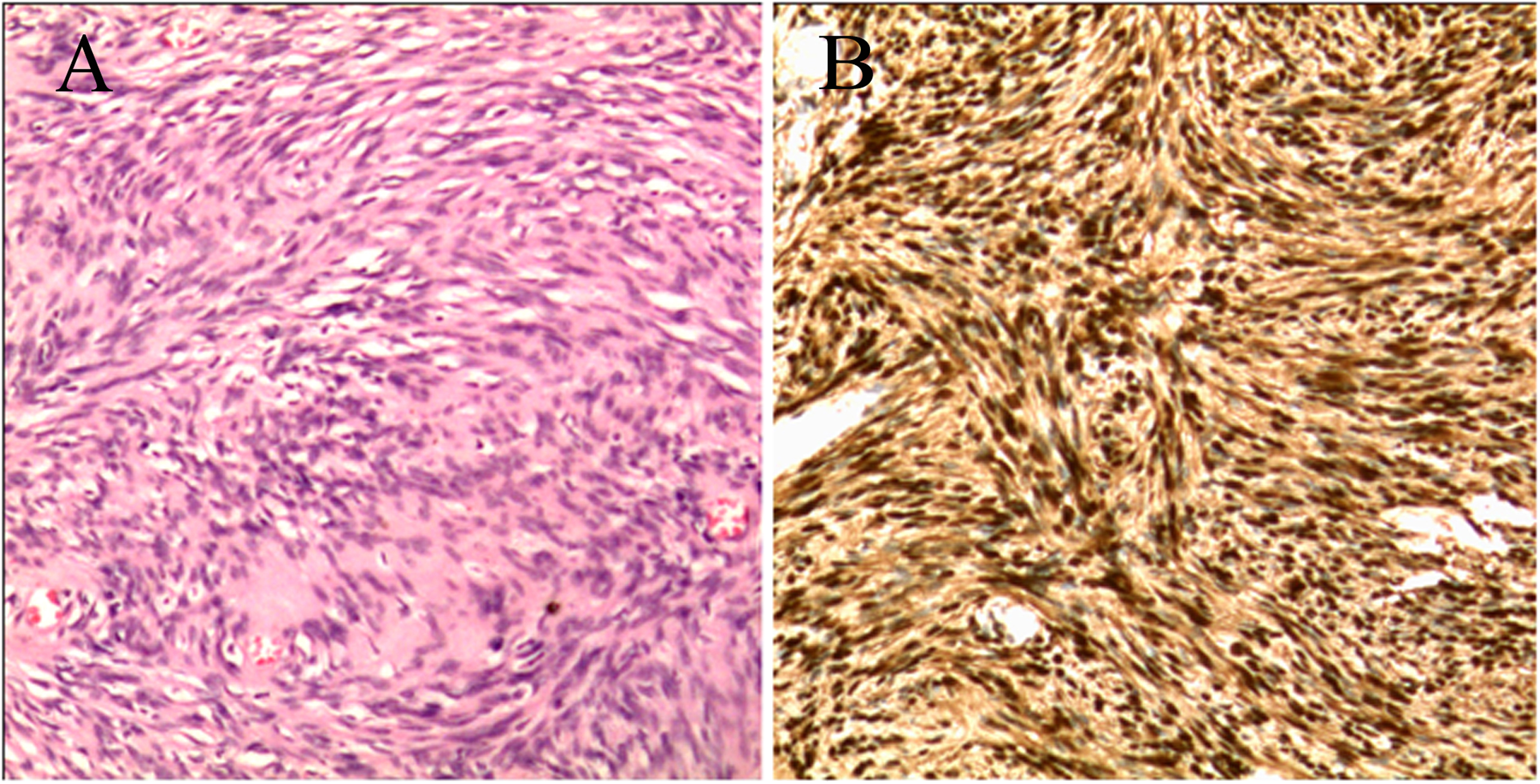

Supplement: Supplementary file 4 — Authors’ original file for figure 4 [file 12891_2014_2282_MOESM4_ESM.tif]

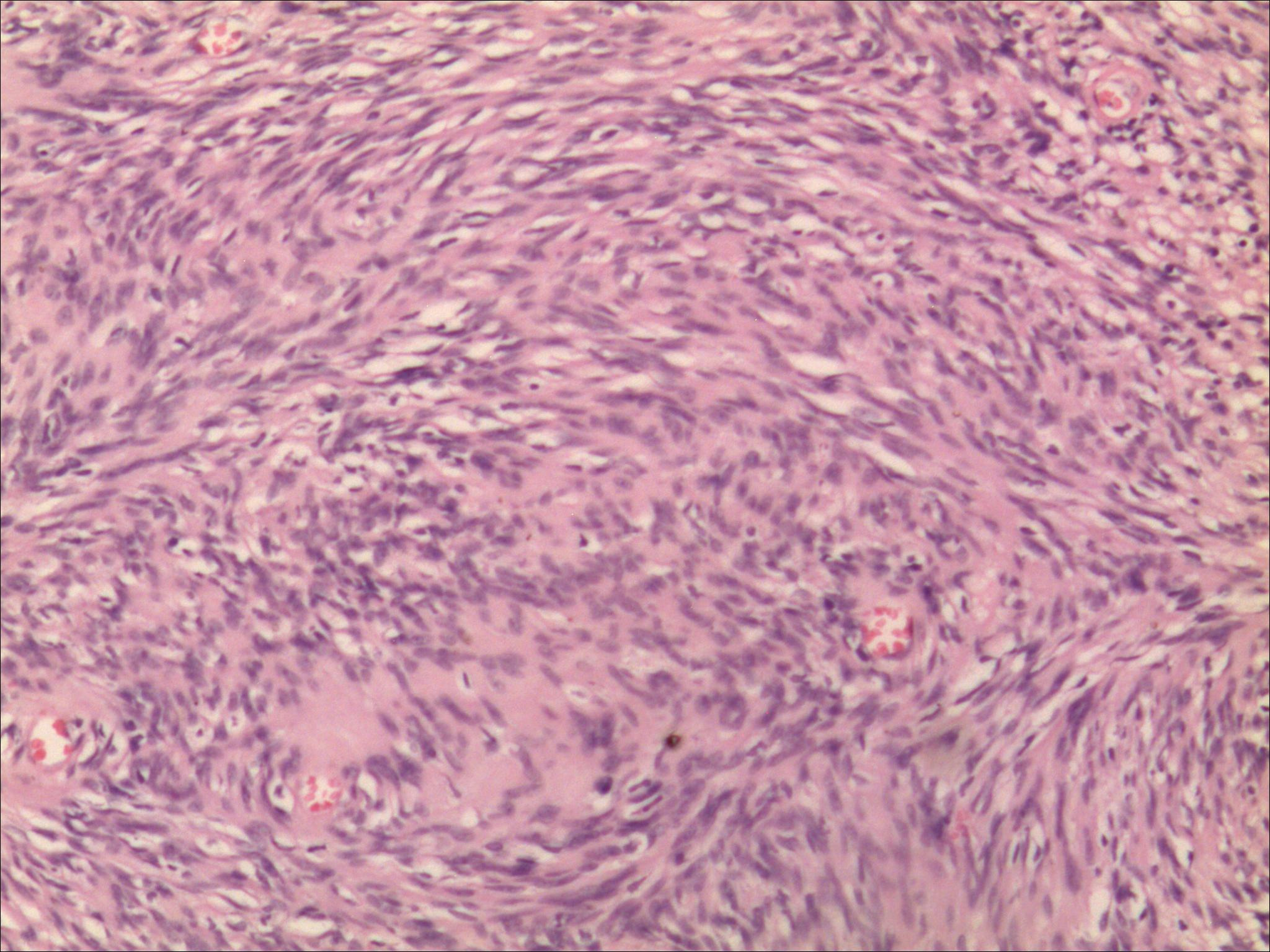

Supplement: Supplementary file 5 — Authors’ original file for figure 6 [file 12891_2014_2282_MOESM5_ESM.jpeg]

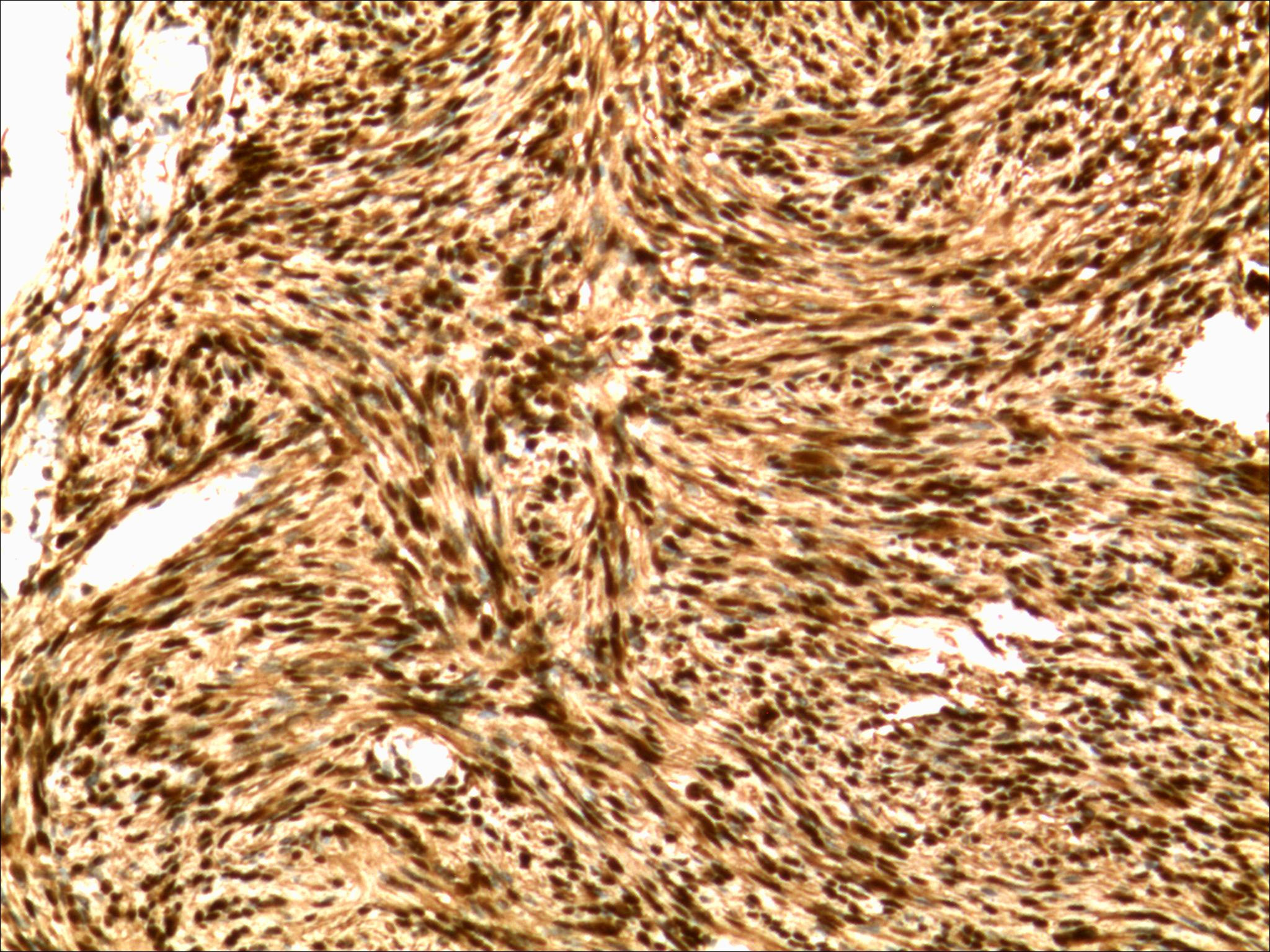

Supplement: Supplementary file 6 — Authors’ original file for figure 7 [file 12891_2014_2282_MOESM6_ESM.jpeg]
